# Supplementary material for: Quality of Life for Head and Neck Cancer Patients: A 10-Year Bibliographic Analysis
Source: Cancers (Basel). 2023 Sep 14;15(18):4551. doi: 10.3390/cancers15184551 (PMC10526491; doi:10.3390/cancers15184551)
Supplement: Supplementary file 1 [file cancers-15-04551-s001.zip › supplementary.pdf]

Quality of Life for Head and Neck Cancer Patients: A 10-Year Bibliographic Analysis

Siti Nur Akmal Ghazali [1], Caryn Mei Hsien Chan [2], Marfu'ah Nik Eezamuddeen [3], Hanani Abdul Manan [4,5] and Noorazrul Yahya [1,*]

[1] Diagnostic Imaging and Radiotherapy, CODTIS, Faculty of Health Sciences, National University of Malaysia, Jalan Raja Muda Aziz, Kuala Lumpur 50300, Malaysia

[2] Clinical Psychology and Behavioural Health Programme, REACH, Faculty of Health Sciences, National University of Malaysia, Jalan Raja Muda Aziz, Kuala Lumpur 50300, Malaysia

[3] Cancer Center MAKNA, Universiti Kebangsaan Malaysia Medical Centre, Cheras, Kuala Lumpur 56000, Malaysia

[4] Functional Image Processing Laboratory, Department of Radiology, Universiti Kebangsaan Malaysia, Cheras, Kuala Lumpur 56000, Malaysia

[5] Department of Radiology and Intervention, Hospital Pakar Kanak-Kanak (Children Specialist Hospital), Universiti Kebangsaan Malaysia, Jalan Yaacob Latif, Bandar Tun Razak, Kuala Lumpur 56000, Malaysia

* Correspondence: azrulyahya@ukm.edu.my; Tel.: +60-3-92897221

Supplementary Items.

List:

Supplementary	Description
**Figure S1**	The journal article production dynamic.
**Figure S2**	The co-citation networks of the journals. The red cluster focused more on psycho-oncology and green cluster much related to oral cancer. The blue cluster much related to oncology.
**Table S1**	The top 10 most impactful articles studies based on total global citations score.
**Table S2**	The top 10 article based on local citations.
**Figure S3**	The co-citation network that shows relationship between cited references works. The center of the figure showed the most repeated cited-reference work which is from Aaronson et al. (1993) [35].
**Figure S4**	The top-10 most productive institutions based on cumulative number of publications.

**Figure S5**	Bibliographic coupling of institutions.
**Table S3**	The number of local citations based on authors
**Figure S6**	The timeline of author's publication since 2013 until 2022
**Figure S7**	The bibliographic coupling of authors. Each node represents the short name of the authors and the distance between two nodes how strong there are related. The cluster is a set of closely related nodes. There were 11 clusters and the most important cluster was red cluster consisted 32 authors
**Figure S8**	The collaboration between countries. Among 38 countries, 16 countries were listed in red cluster, 12 in green cluster and 7 in blue cluster
**Figure S9**	a) Distribution the number of publications produced based on country, b) The distribution of estimated age-standardized mortality rates of head and neck cancer patients around the world. Data from Globocan.com, c) The prevalence of head and neck cancer in 5 years among all population in the world. Reprinted from International Agency For Research On Cancer, 'Cancer today - Data visualization tools for exploring the global cancer burden in 2020', Copyright 2022, http://gco.iarc.fr/today (accessed February 2023). The map was generated using the GLOBOCAN website mapping tool (https://gco.iarc.fr/today/online-analysis-map) by selecting the 'lip, oral cavity', 'oropharynx', 'hypopharynx', 'larynx', 'salivary gland' and 'nasopharynx' cancer sites. Estimated age-standardized rates of head and neck cancer mortality rates and number of prevalence (5-years) worldwide are shown for both sexes and all ages.
**Figure S10**	The tree plot represents the number of occurrence and percentage of author's used keywords.
**Figure S11**	The field three plot showed that radiotherapy keyword was used by three authors and none of the interested in depression.
**Figure S12**	The emergence of each keywords from first phase to second phase.

Figure S1: THE JOURNAL ARTICLE PRODUCTION DYNAMIC.

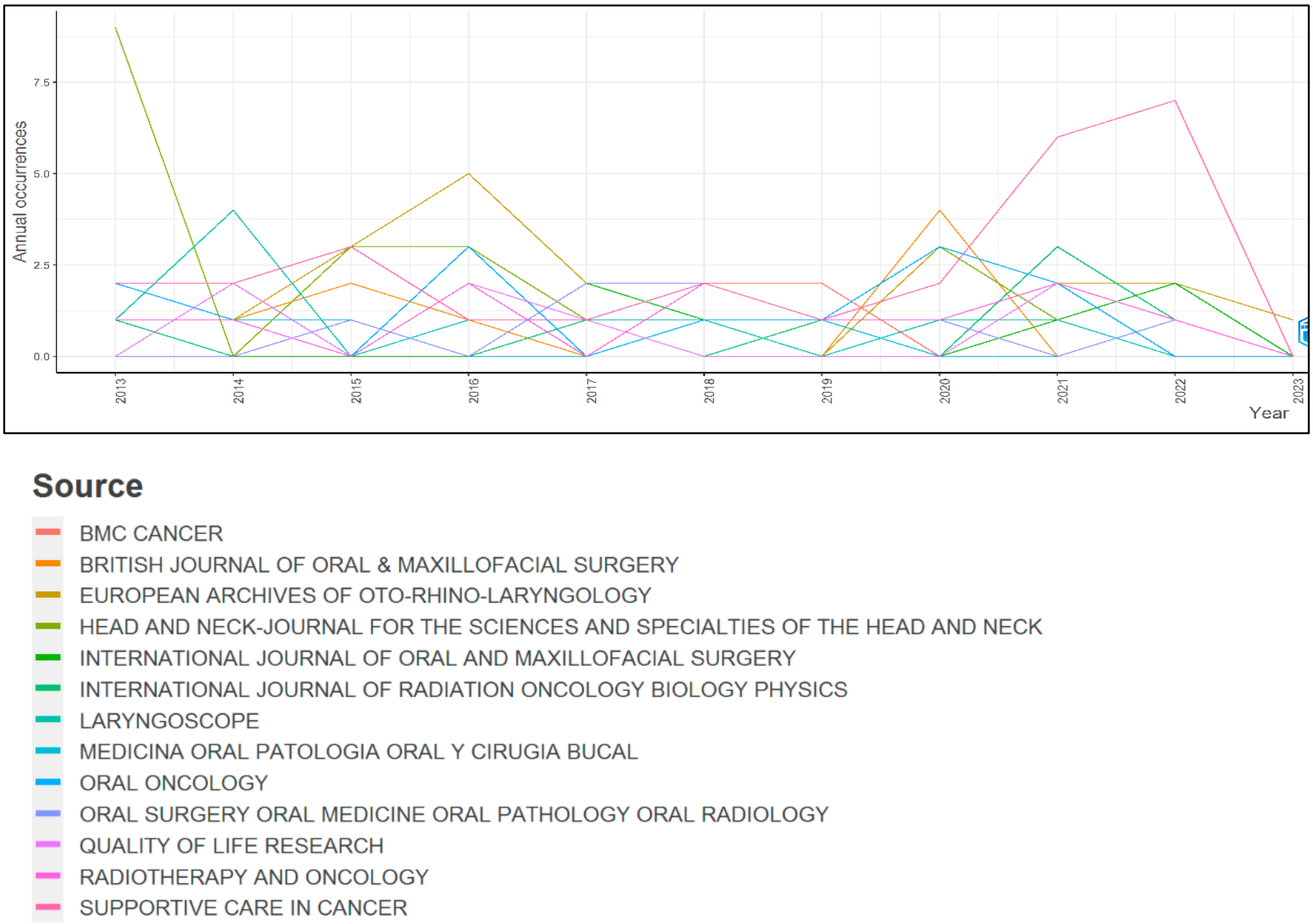

Figure S2: THE CO-CITATION NETWORKS OF THE JOURNALS

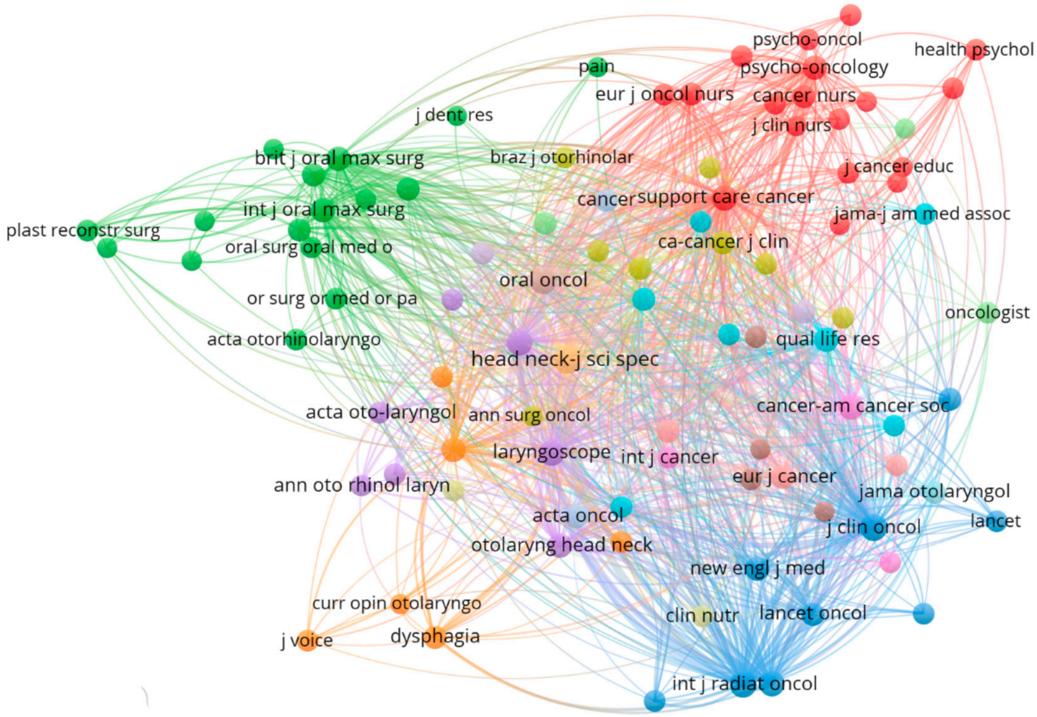

Note: The red cluster focused more on psycho-oncology and green cluster much related to oral cancer. The blue cluster much related to oncology.

Table S1: THE TOP 10 MOST IMPACTFUL ARTICLES STUDIES BASED ON TOTAL GLOBAL CITATIONS SCORE.

Rank	Paper	DOI	Total Global Citations	Total Citations per Year
1	Dziegielewski P. T (2013)	10.1001/jamaoto.2013.2747	157	14.27
2	Hunter K. U. (2013)	10.1016/j.ijrobp.2012.08.030	134	12.18
3	Langius J. A. E. (2013)	10.1080/01635581.2013.741749	93	8.45
4	Pauli N. (2013)	10.3109/0284186X.2012.744466	83	7.55
5	Gellrich N. C (2015)	10.3390/nu7042145	70	7.78
6	Deng J. (2013)	10.1002/hed.23084	69	6.27
7	Verdonck-De Leeuw I. M. (2014)	10.1016/j.radonc.2014.01.002	68	6.80
8	Pottel L. (2014)	10.1111/ecc.12179	67	6.70
9	Oskam I. M. (2013)	10.1016/j.oraloncology.2012.12.005	67	6.09
10	Vainshtein J. M. (2015)	10.1016/j.ijrobp.2014.12.045	64	7.11

Table S2: THE TOP 10 ARTICLE BASED ON LOCAL CITATIONS.

Document	Local Citations	Global Citations	LC/GC Ratio (%)	Normalized Local Citations	Normalized Global Citations
Verdonck-De Leeuw I. M, (2014)	20	68	29.41	5.98	2.67
De Melo M. R (2013)	16	46	34.78	3.97	1.39
Oskam I. M (2013)	14	67	20.90	3.48	2.02
Hunter K. U (2013)	13	134	9.70	3.23	4.04
Singer S (2015)	13	43	30.23	5.02	2.15
Boyapati R.P (2013)	11	25	44.00	2.73	0.75
Chen Am (2014)	10	51	19.61	2.99	2.00
Liao L. J. (2019)	10	31	32.26	7.14	2.89
Bottomley A. (2014)	9	29	31.03	2.69	1.14
Barrios R. (2015)	9	25	36.00	3.48	1.25

Figure S3: THE CO-CITATION NETWORK THAT SHOWS RELATIONSHIP BETWEEN CITED-REFERENCES WORKS.

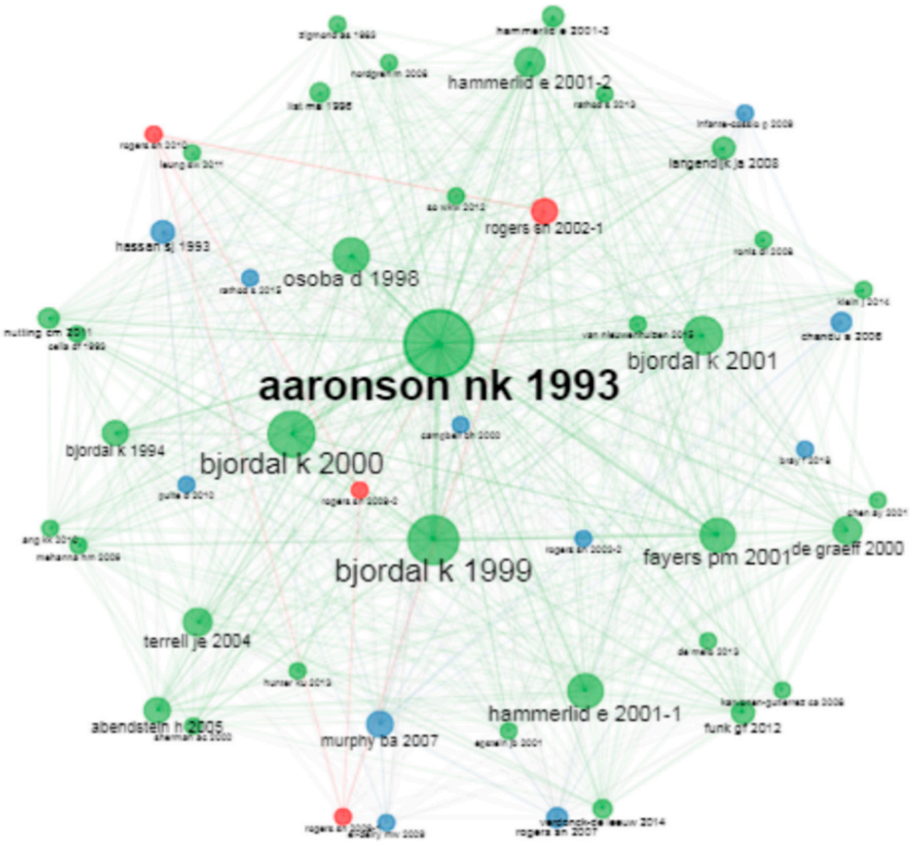

Note: The center of the figure showed the most repeated cited-reference work which is from Aaronson et al. (1993) [35].

Figure S4: THE TOP-10 MOST PRODUCTIVE INSTITUTIONS BASED ON CUMULATIVE NUMBER OF PUBLICATIONS.

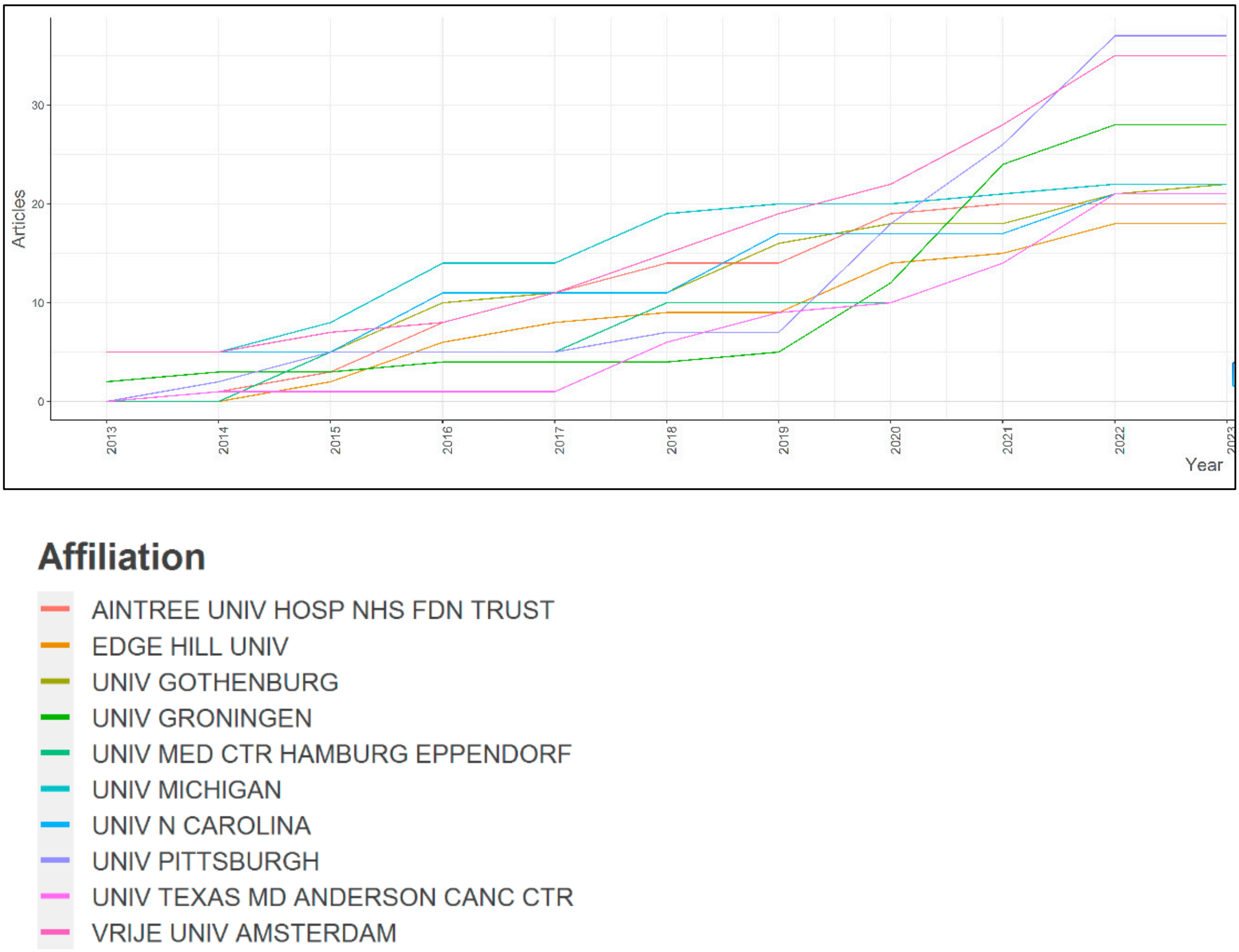

Figure S5: BIBLIOGRAPHIC COUPLING OF INSTITUTIONS

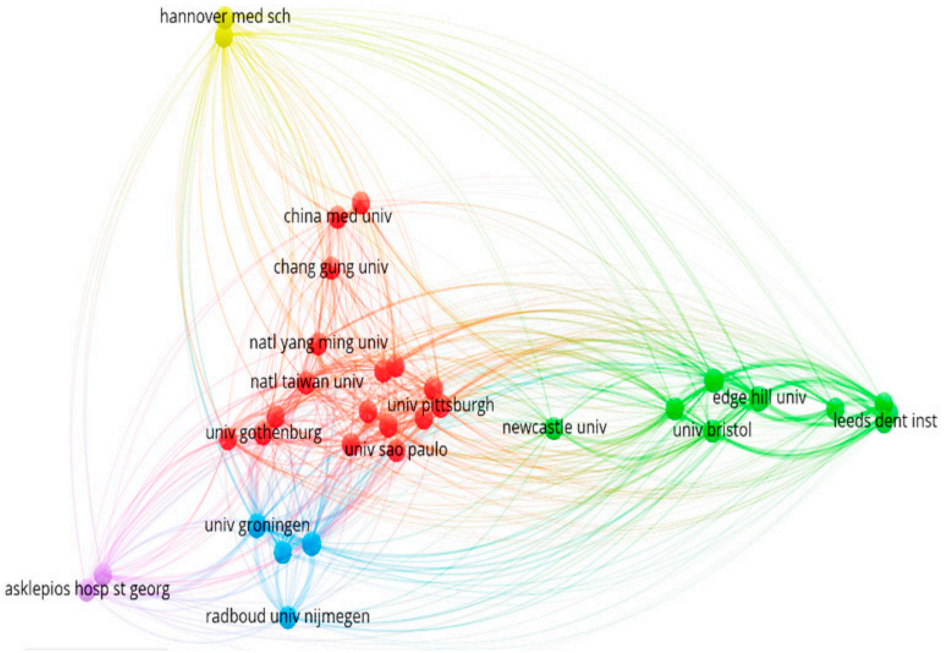

Table S3: THE NUMBER OF LOCAL CITATIONS BASED ON AUTHORS.

Rank	Author	Local Citations
1	Leemans C.R.	57
2	Langendijk J.A.	51
3	Verdonck-De Leeuw I. M.	47
4	Rogers S. N.	40
5	De Bree R.	37
6	Doornaert P.	37
7	Aaronson N. K	34
8	Eisbruch A.	26
9	Singer S.	25
10	Lowe D.	24
11	Barrios R.	22
12	Benezery K.	22
13	Bozec A.	22
14	Bravo M.	22
15	Chamorey E.	22
16	Dassonville O.	22
17	Hammerlid E.	22
18	Licitra L.	22
19	Peyrade F.	22
20	Poissonnet G.	22
21	Buffart L. M.	21
22	Chepeha D. B.	21
23	Feng F. Y.	21
24	Buter J.	20
25	Heymans M. W.	20
26	Rietveld D. H.	20
27	Slotman B. J.	20
29	Santini J.	19
30	Semple C.	18

Figure S6: THE TIMELINE OF AUTHOR'S PUBLICATION SINCE 2013 UNTIL 2022

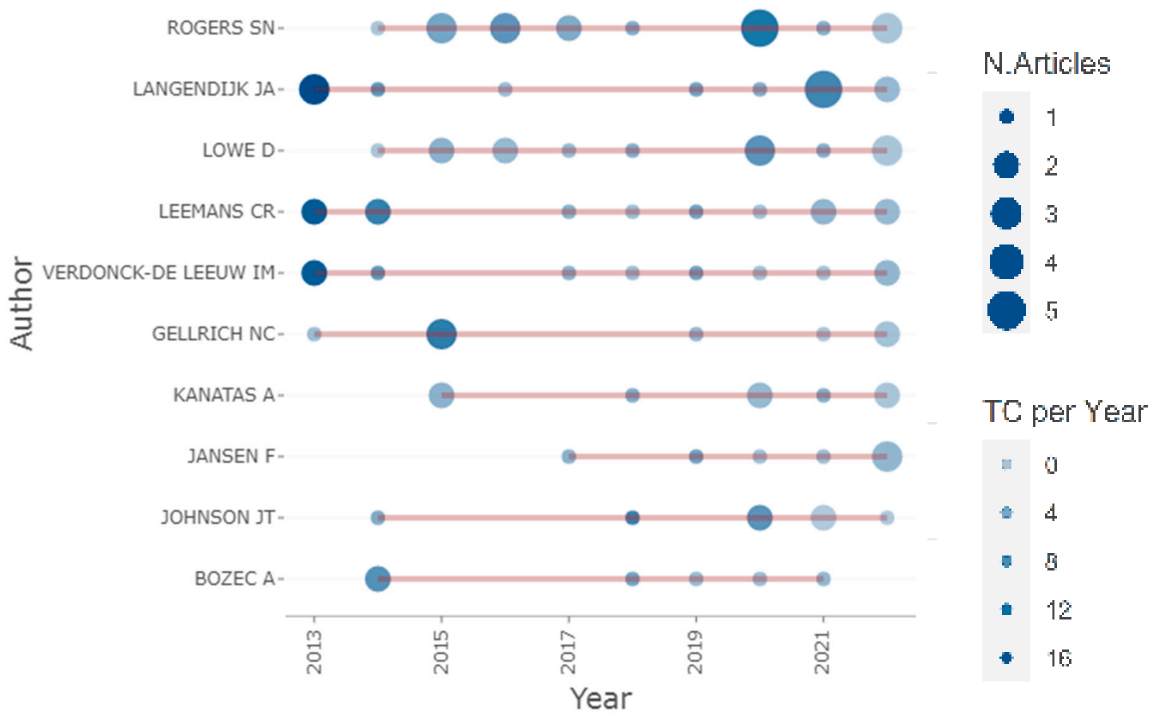

Figure S7: THE BIBLIOGRAPHIC COUPLING OF 103 AUTHORS

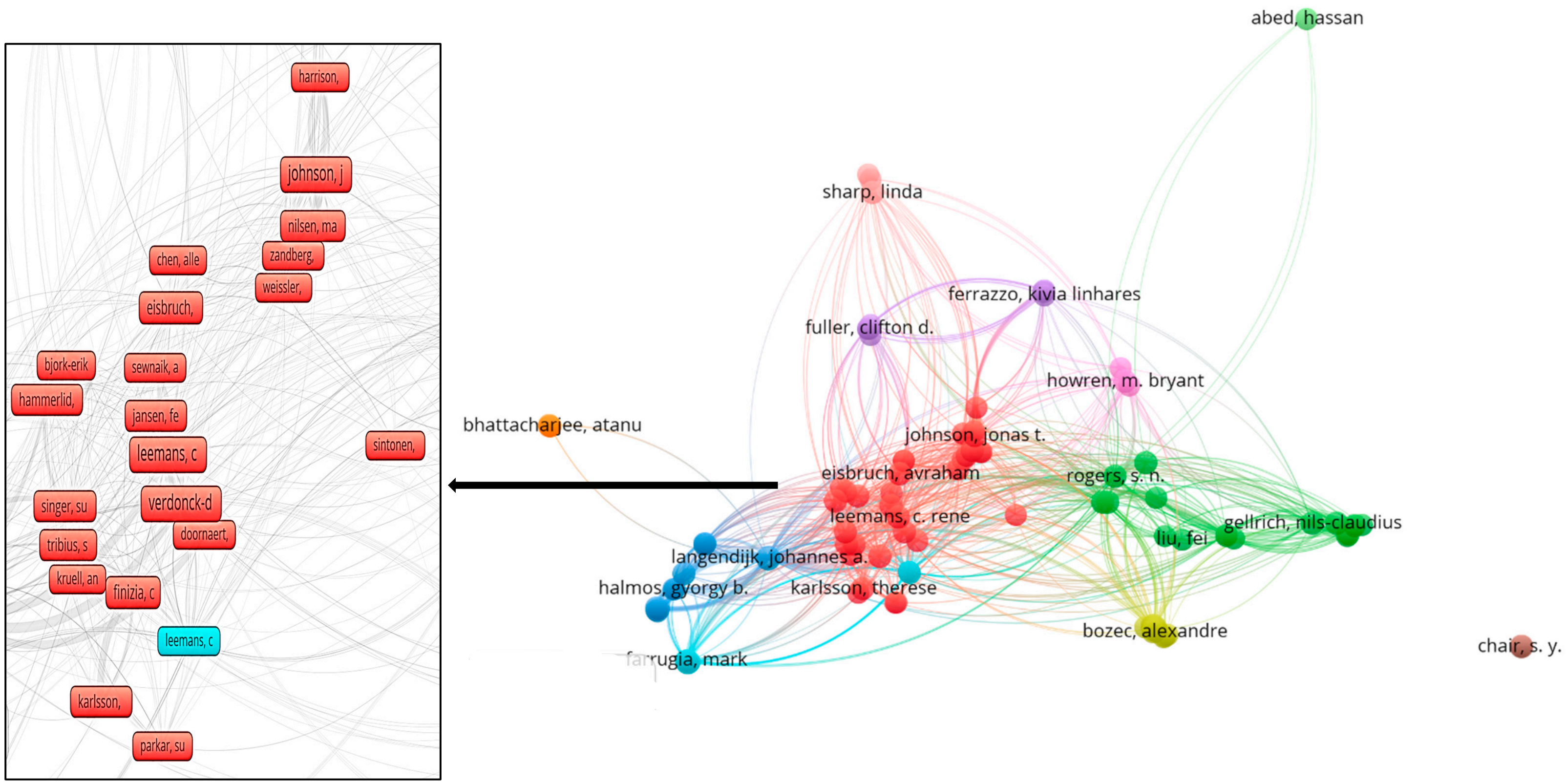

Note: Each node represents the short name of the authors and the distance between two nodes how strong there are related. Resolution set to 0.5. The cluster is a set of closely related nodes. There were 11 clusters and the most important cluster was red cluster consisted 32 authors (only 21 was shown in figure on the left).

Figure S8: THE COLLABORATION BETWEEN COUNTRIES

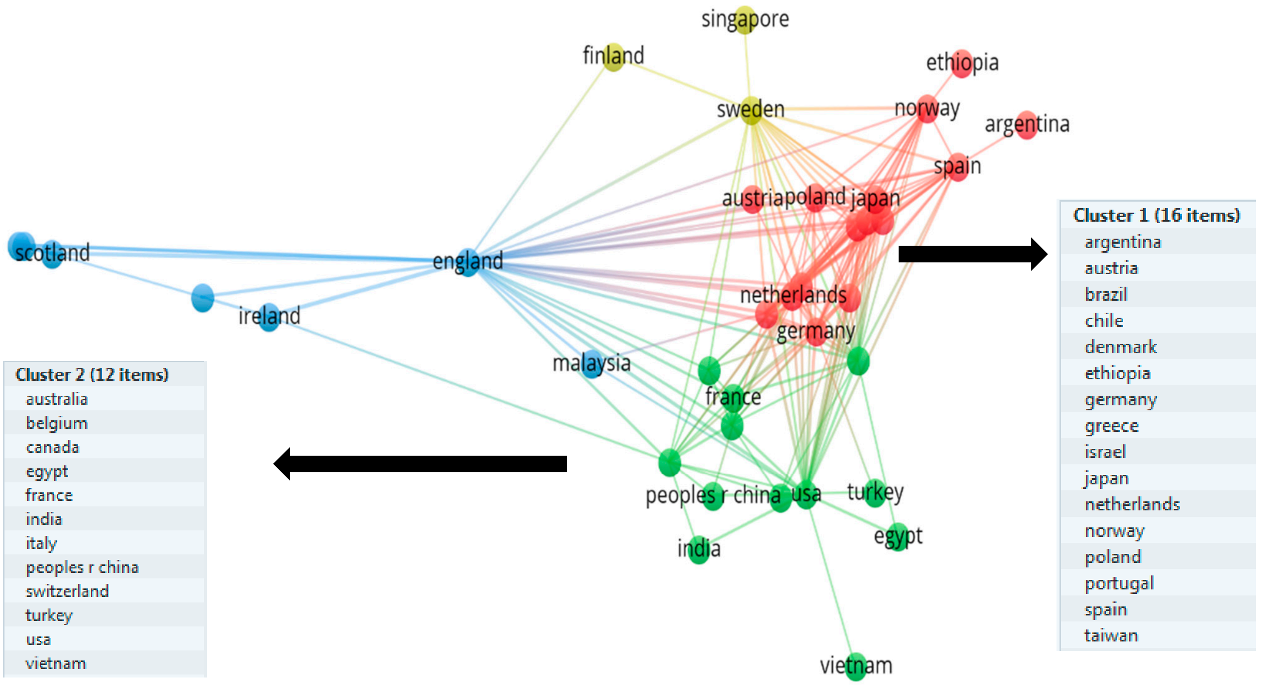

Note: Among 38 countries, 16 countries was listed in red cluster, 12 in green cluster and 7 in blue cluster. There are 4 clusters.

Figure S9: GLOBACON DATA vs NUMBER OF PUBLICATIONS

(a)

(b)

(c)

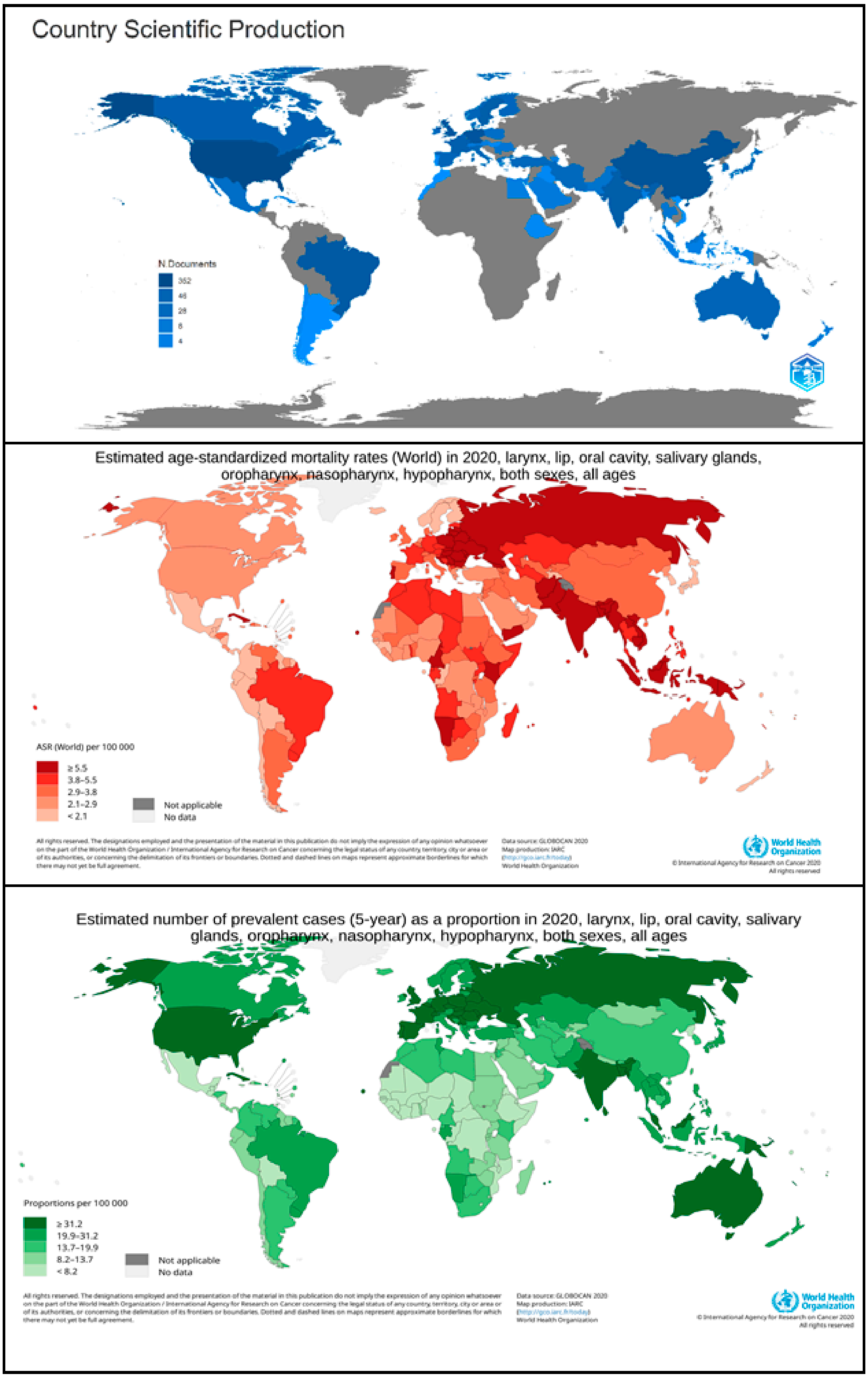

a) Distribution the number of publications produced based on country, b) The distribution of estimated age-standardized mortality rates of head and neck cancer patients around the world. Data from Globocan.com, c) The prevalence of head and neck cancer in 5 years among all population in the world. Reprinted from International Agency for Research On Cancer, 'Cancer today - Data visualization tools for exploring the global cancer burden in 2020', Copyright 2022, http://gco.iarc.fr/today (accessed February 2023). The map was generated using the GLOBOCAN website mapping tool (https://gco.iarc.fr/today/online-analysis-map) by selecting the 'lip, oral cavity', 'oropharynx', 'hypopharynx', 'larynx', 'salivary gland' and 'nasopharynx' cancer sites. Estimated age-standardized rates of head and neck cancer mortality rates and number of prevalence (5-years) worldwide are shown for both sexes and all ages.

Figure S10: THE NUMBER OF OCCURANCE AND PERCENTAGE OF THE KEYWORDS

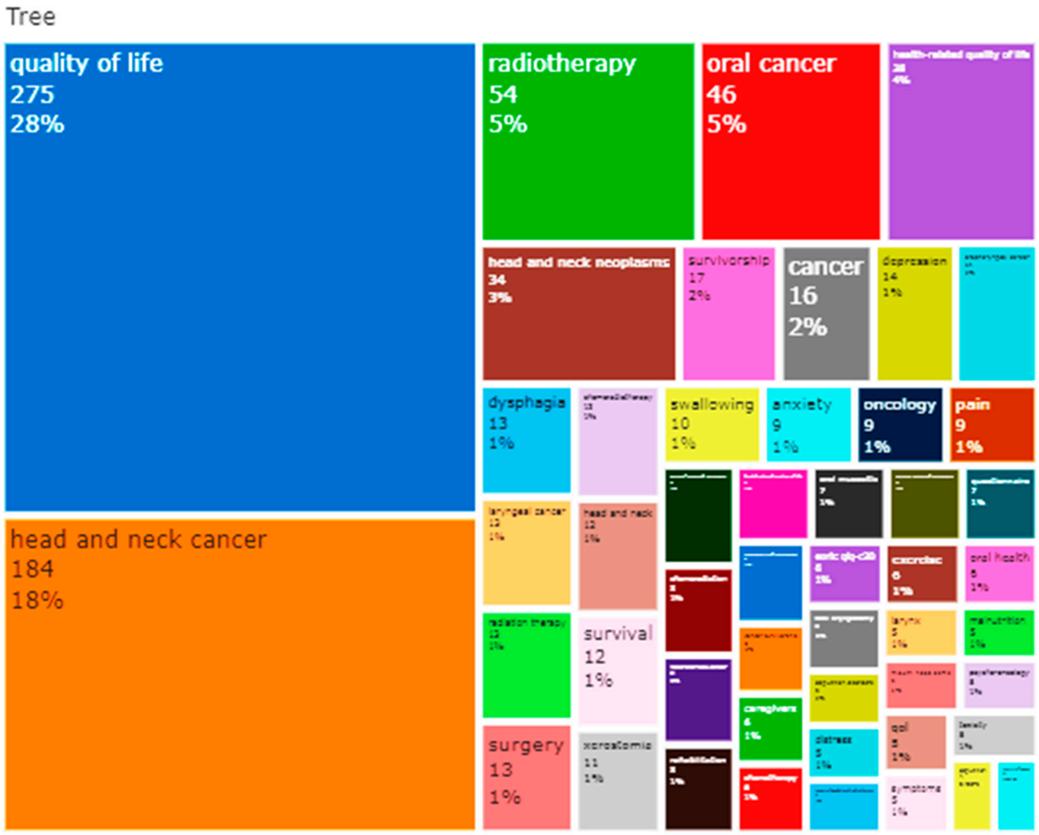

Note: The tree plot represent the number of occurance and percentage of the keywords over the 50 keywords. The selected keywords are top 50 the keywords with highest coccurance. Refer table below for list:

Rank	Authors keywords	Occurrences
1	Quality of life	275
2	Head and neck cancer	184
3	Radiotherapy	54
4	Oral cancer	46
5	Health-related quality of life	38
6	Head and neck neoplasms	34
7	Survivorship	17
8	Cancer	16
9	Depression	14
10	Oropharyngeal cancer	14

11	Dysphagia	13
12	Laryngeal cancer	13
13	Radiation therapy	13
14	Surgery	13
15	Chemoradiotherapy	12
16	Head and neck	12
17	Survival	12
18	Xerostomia	11
19	Swallowing	10
20	Anxiety	9
21	Oncology	9
22	Pain	9
23	Patient-reported outcomes	9
24	Chemoradiation	8
25	Head-and-neck cancer	8
26	Rehabilitation	8
27	Health related quality of life	7
28	Oral mucositis	7
29	Patient reported outcomes	7
30	Questionnaire	7
31	Squamous cell carcinoma	7
32	Cancer survivorship	6
33	Caregivers	6
34	Chemotherapy	6
35	EORTC QLQ-c30	6
36	Exercise	6
37	Oral health	6
38	Total laryngectomy	6
39	Deglutition disorders	5
40	Distress	5
41	Intensity-modulated radiotherapy	5
42	Larynx	5
43	Malnutrition	5
44	Mouth neoplasms	5
45	Psycho-oncology	5
46	QOL	5
47	Symptoms	5
48	Toxicity	5
49	Deglutition	4
50	Eortc QLQ-H&N35	4

Figure S11: KEYWORDS VS TOP JOURNALS AND TOP AUTHORS

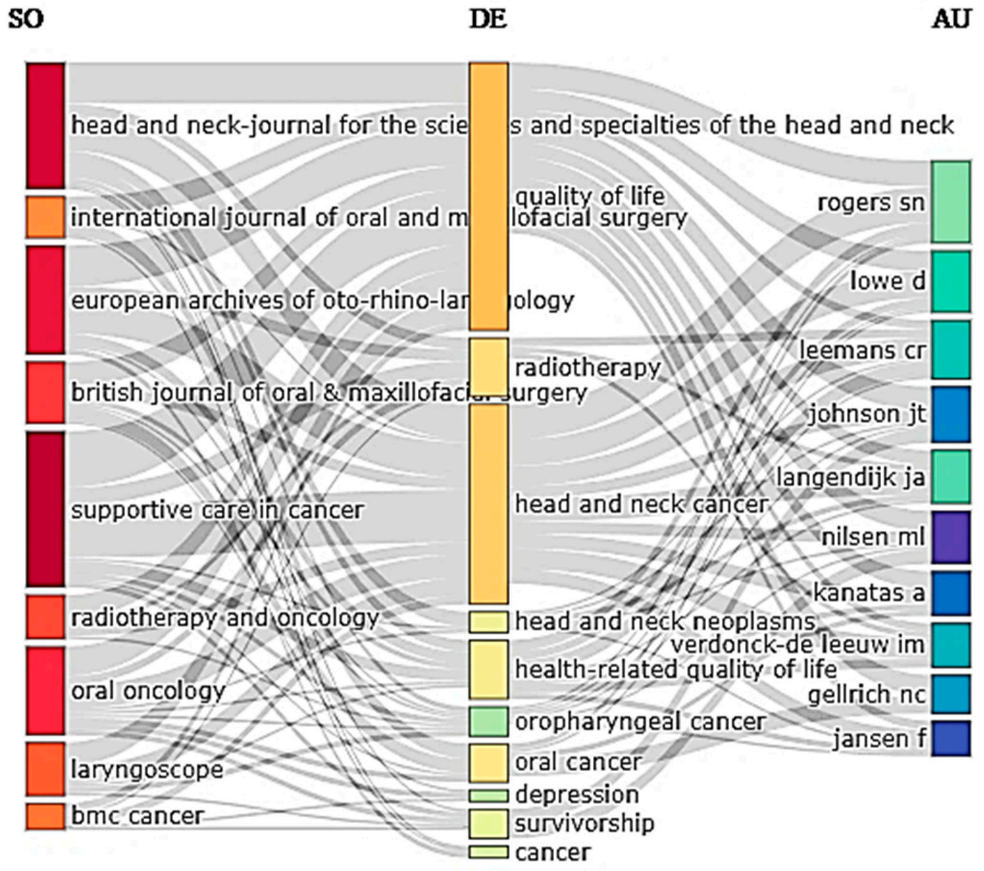

The field three plot showed that survivorship was studied by Langendijk J.A and Nilsen M.L.

Figure S12: KEYWORDS DEVELOPMENT

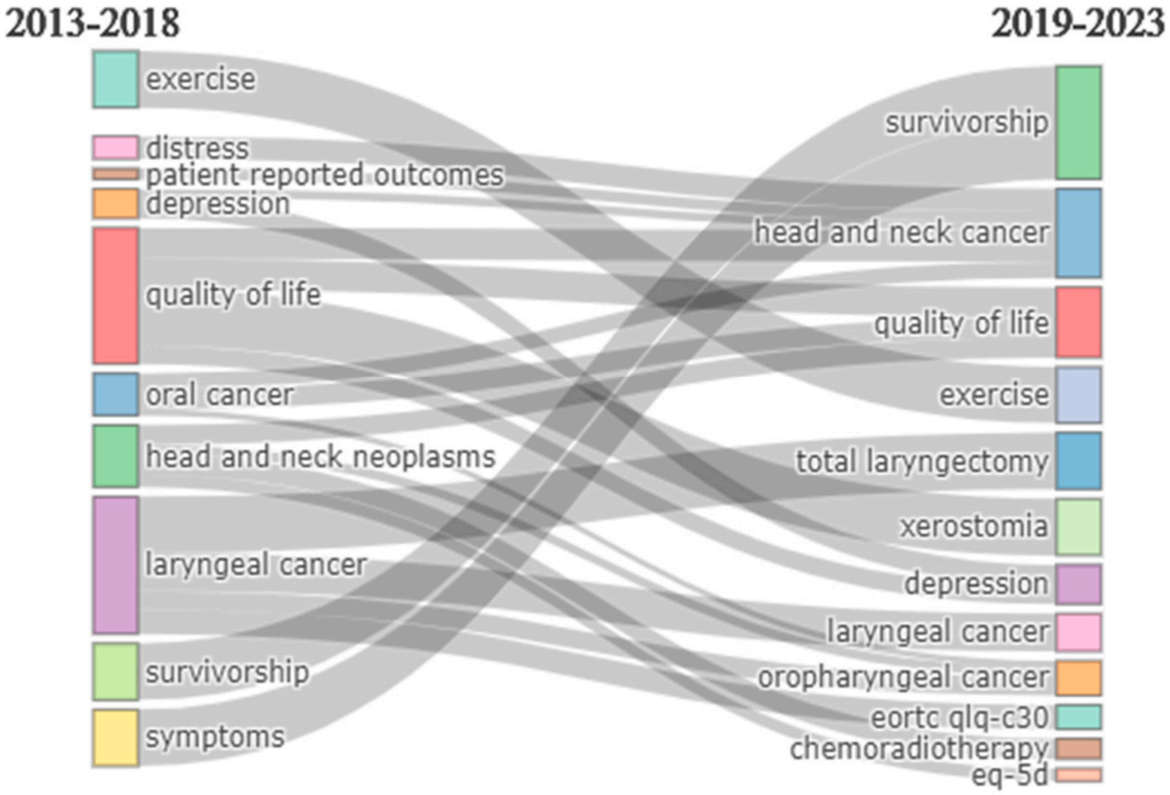

The emergence of each keywords from first phase to second phase.

35. Aaronson, N. K., Ahmedzai, S., Bergman, B., Bullinger, M., Cull, A., Duez, N. J., Filiberti, A., Flechtner, H., Fleishman, S. B., De Haes, J. C. & Et Al. 1993. The European Organization for Research and Treatment of Cancer QLQ-C30: a quality-of-life instrument for use in international clinical trials in oncology. *J Natl Cancer Inst* 85(5): 365-376.
